# 2157. Phase 1 Study in Healthy Chinese Subjects of Novel Prodrug, MRX-4

**DOI:** 10.1093/ofid/ofad500.1780

**Published:** 2023-11-27

**Authors:** Haijing Yang, Size Li, Hailin Wang, Guoying Cao, Jing Zhang

**Affiliations:** Huashan Hospital of fudan university, Shanghai, Shanghai, China; Huashan Hospital, Shanghai, Shanghai, China; Shanghai MicuRx Pharmaceutical Co., Ltd., Shanghai, Shanghai, China; Phase Ⅰ Clinical Research Center, Huashan Hospital, Fudan University, Shanghai, Shanghai, China; Phase Ⅰ Clinical Research Center, Huashan Hospital, Fudan University, Shanghai, Shanghai, China

## Abstract

**Background:**

MRX-4 is a prodrug of contezolid (MRX-1), a new oxazilidone approved in China. We evaluated the safety and pharmacokinetic (PK) profile of IV and oral MRX-4 and its metabolites.

**Methods:**

This randomized, double-blind, placebo-controlled study was conducted at Huashan Hospital, (Shanghai, China) with four-arm design: a) IV, single-dose escalation group (500 mg, 1000 mg, 1500 mg, or 2000 mg); b) IV, repeated-dose group (2000 mg loading dose followed by 1000 mg maintenance dose, q12 h for 11 consecutive days using 1-1.5 hr infusion time); c) oral, single-dose escalation group (500 mg or 1500 mg); d) oral, repeated-dose group (1500 mg, q12 h for 11 consecutive days). Safety evaluation was conducted in each dose group.

The blood and urine concentrations of MRX-4, MXR-1, and the inactive metabolites (MRX-1352) were measured, using high-performance liquid chromatography-tandem mass spectrometry (HPLC-MS/MS).

**Results:**

A total of 70 subjects completed the trial. MRX-4 concentration was not possible to measure due to rapid hydrolisis into MRX-1352. In the MRX-4 group, 67 treatment-emergent adverse events (TEAEs) were observed in 27 of 55 subjects (49%); 24 TEAEs occurred in 14 subjects in the IV group (n = 36) and 43 TEAEs in 13 subjects in the oral group (n = 19). Six adverse drug reactions (ADRs) were observed in 4 subjects in the IV group, which were predominantly a mild transient decrease in white blood cells that self-resolved. In the oral group, 23 ADRs were observed in 9 of 19 subjects (47%), primarily nausea and vomiting that self-resolved in the 1500 mg repeated-dose group. No decrease in platelet count was observed in any dose group. PK parameters of the inactive intermediate metabolite (MRX-1352), and the active metabolite (MRX-I, contezolid) are shown:

**Figure:**
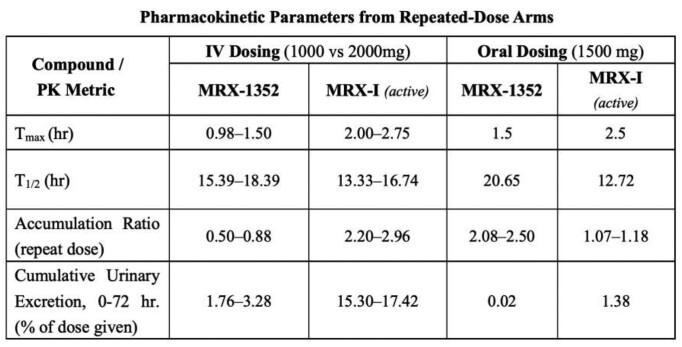

**Figure:**
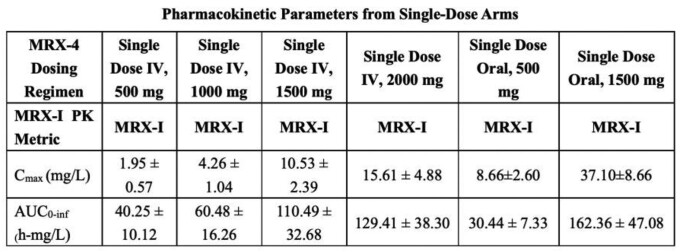

**Conclusion:**

MRX-4 was safe and well tolerated among Chinese healthy subjects, with treatment adverse events being mild and transitory. MRX-4, administered via IV or oral, demonstrated rapid and complete conversion to contezolid. PK parameters support the potential for use of MRX-4 as the IV companion to oral contezolid.

**Disclosures:**

**All Authors**: No reported disclosures

